# Retraction: Optimizing the impact of time domain segmentation techniques on upper limb EMG decoding using multimodal features

**DOI:** 10.1371/journal.pone.0346389

**Published:** 2026-04-08

**Authors:** The PLOS One Editors

The *PLOS One* Editors retract this article [1] due to concerns about:

- Compromised peer review.
- Compliance with PLOS policy on Human Subjects Research as the ethics approval document issued by the National University of Sciences and Technology (NUST) that was provided before publication indicates that approval for data collection was granted by a member of the author list.
- The inclusion of insufficient information on data handling/processing and methodology to enable reproducibility.

In response to the concerns regarding compliance with Human Subjects Research policy, the corresponding author stated that COMSATS approved the study and that COMSATS granted an exemption for approval, and provided supporting documentation. The Editors do not consider the additional documentation sufficient to resolve this issue, and remain concerned regarding the discrepancy between the source of ethics approval declared in the article and post-publication responses.

All authors did not agree with the retraction.
